# Contribution of IL-17 in Steroid Hyporesponsiveness in Obese Asthmatics Through Dysregulation of Glucocorticoid Receptors α and β

**DOI:** 10.3389/fimmu.2020.01724

**Published:** 2020-08-04

**Authors:** Saba Al Heialy, Mellissa Gaudet, Rakhee K. Ramakrishnan, Andrea Mogas, Laila Salameh, Bassam Mahboub, Qutayba Hamid

**Affiliations:** ^1^College of Medicine, Mohammed Bin Rashid University of Medicine and Health Sciences, Dubai, United Arab Emirates; ^2^Meakins-Christie Laboratories, Research Institute of the McGill University Healthy Center, Montreal, QC, Canada; ^3^Sharjah Institute for Medical Research, College of Medicine, University of Sharjah, Sharjah, United Arab Emirates; ^4^Pulmonary Medicine Department, Rashid Hospital, Dubai, United Arab Emirates

**Keywords:** asthma, obesity, IL-17, steroid hyporesponsiveness, glucocorticoid receptors, inflammation

## Abstract

Obesity is on the rise worldwide and is one of the most common comorbidities of asthma. The chronic inflammation seen in obesity is believed to contribute to this process. Asthma and obesity are associated with a poorer prognosis, more frequent exacerbations, and poor asthma control to standard controller medication. Difficult-to-treat asthma is associated with increased levels of Th17 cytokines which have been shown to play a central role in the upregulation of glucocorticoid receptor-beta (GR-β), a dominant-negative inhibitor of the classical GR-α. In this study, we studied the role of IL-17 cytokines in steroid hyporesponsiveness in obese asthmatics. We stimulated lean and obese adipocytes with IL-17A and IL-17F. Adipocytes obtained from obese patients cultured *in vitro* in the presence of IL-17A for 48 h showed a decrease in GRα/GRβ ratio as compared to adipocytes from lean subjects where GR-α/GR-β ratio was increased following IL-17A and IL-17F stimulation. At protein level, GR-β was increased in obese adipocytes with IL-17A and IL-17F stimulation. IL-8 and IL-6 expression was increased in IL-17-stimulated obese adipocytes. Pre-incubation with Dexamethasone (Dexa) led to a decrease in GR-α/GR-β ratio in obese adipocytes which was further affected by IL-17A whereas Dexa led to an increase in GR-α/GR-β ratio in lean adipocytes which was decreased in response to IL-17A. TGF-β mRNA expression was decreased in obese adipocytes in response to Th17 cytokines. We next sought to validate these findings in obese asthmatic patients. Serum obtained from obese asthmatic subjects showed a decrease in GRα/GRβ protein expression with an increase in IL-17F and IL-13 as compared to serum obtained from non-obese asthmatics. In conclusion, steroid hyporesponsiveness in obese asthmatic patients can be attributed to Th17 cytokines which are responsible for the dysregulation of the GRα/GRβ ratio and the inflammatory response.

## Introduction

Worldwide, incidence of obesity is on the rise at an alarming rate. It is estimated that by 2030, 38% of the world's population will be overweight and 20% will be obese (1). Obesity is simply defined as excess weight for height where the body mass index (BMI) is equal or greater to 30 kg/m^2^ Obesity is associated with a multitude of metabolic abnormalities ranging from diabetes to cardiovascular disease and asthma (2, 3). It is believed that these associations are due mostly in part to the chronic inflammation associated with obesity. High levels of pro-inflammatory cytokines, such as interleukin (IL)-6 (4), IL-8 (5), and Tumor Necrosis Factor-alpha (TNF-α) (6) are seen in various models of obesity. Adipose tissue, which is composed mainly of adipocytes, is now recognized as an organ which contributes to systemic inflammation (7). Adipose explants from obese patients show an increase in mediators such as IL-6, TNF-α, angiotensinogen and complement C3 (8). Low-grade systemic inflammation has been shown to regulate adipogenesis and insulin resistance (9).

As adipose tissue has been shown to contribute to high levels of serum IL-6, this has prompted recent studies to focus on the role of Th17 cells in obesity. IL-6 is necessary for the polarization of CD4+ T cells into Th17 cells (10). The main role of Th17 cells is to clear bacteria and fungi. However, beyond their protective role, Th17 cells are implicated in many inflammatory conditions and are the major cellular source of IL-17 cytokines, most notably IL-17A and IL-17F (11). IL-17 has been shown to be upregulated in obese subjects (12). In a mouse model of diet-induced obesity, IL-17A production was enhanced by CD4+ T cells. Moreover, IL-17 is an important player in severe asthma (13). IL-17A and IL-17F production is increased with severity of the disease and Th17 cells are now recognized as the major T helper subset in severe asthma (14). The presence of IL-17 is crucial due to its role in steroid resistance through the dysregulation of glucocorticoid receptors.

In the United States, ~60% of patients with severe asthma are obese (15). Asthma is a heterogeneous disease defined by many phenotypes. Understanding the mechanisms underlying the various asthma phenotypes is important in predicting therapy. Asthma associated with obesity is a complex phenotype which is characterized by worsening outcomes such as poor control and increased exacerbations akin to severe asthma (16). Overweight asthmatic children show a decreased response to inhaled budesonide compared to normal weight asthmatic children (17). An increasing body of literature show a reduction in steroid responsiveness in obese asthmatics compared to their lean counterparts (18, 19). Steroid hyporesponsiveness is one of the major characteristics of severe asthma and makes treatment of symptoms challenging. A study by Vazquez-Tello et al. showed that IL-17 cytokine stimulation of peripheral blood mononuclear cells (PBMCs) leads to an upregulation of the glucocorticoid receptor-beta (GR-β) (20). Alternative splicing of the GR transcript generates two isoforms of GR: GR-α and GR-β. GR-β is a dominant negative-regulator of the active GR-α and has been associated with steroid hyporesponsiveness.

Although studies have already shown a positive correlation between IL-17 and the inflammatory conditions of asthma and obesity individually, no studies to our knowledge have looked at the role of IL-17 in obese asthmatics. Moreover, the mechanism underlying the decreased responses to steroid in obese asthmatics has not been fully elucidated. We hypothesized that IL-17 cytokines are involved in steroid resistance described in obese asthmatics through the dysregulation of GR-α and GR-β.

## Materials and Methods

### Pre-adipocyte & Adipocyte Cell Culture and Treatment

Subcutaneous human pre-adipocyte from lean and obese subjects were purchased from ATCC (VA, USA) & ZenBio (NC, USA). Table 1 shows the data on pre-adipocytes obtained from lean and obese subjects. Pre-adipocytes were cultured in DMEM/Ham's F-12 (1:1, v/v) media (Thermo Fisher Scientific Inc., Pittsburgh, PA, USA) supplemented with: 0.01M HEPES pH 7.4 (Thermo Fisher Scientific Inc.), 10% fetal bovine serum and 100 U/ml penicillin/streptomycin. Pre-adipocytes were grown to 80% confluence in 10 cm dishes (Corning Inc., Corning, NY, USA) and detached using 0.05% Trypsin-EDTA (Thermo Fisher Scientific Inc.) and seeded in 6 well plates (Sigma-Aldrich, Ontario, Canada). Once confluency reached, the differentiation process was started using Adipocyte Differentiation Medium (ZenBio, NC,USA) for 7 days. The differentiation media was then changed to Adipocyte Maintenance Medium (ZenBio) as detailed in the Subcutaneous Human Adipocyte Manual ZBM0001.05 from ZenBio. Mature adipocytes were kept in culture for no longer than 2 weeks post differentiation.

**Table 1:** Data of adipocytes from lean and obese subjects.

	**Lean adipocytes**	**Obese adipocytes**
N	4	3
Age, year	37.8 ± 9.9	46 ± 4.4
BMI, kg/m^2^	20.6 ± 2.2	33.4 ± 3.5

*BMI, body mass index. Values shown are mean ± SE*.

Mature adipocytes were starved with DMEM/Ham's F-12 (1:1, v/v) media supplemented with: 0.01 M HEPES pH 7.4, 0.5% fetal bovine serum and 100 U/ml penicillin/streptomycin overnight. Cells were then stimulated with recombinant human IL-17A and F cytokines (100 ng/ml; R&D systems, Minneapolis, MN, USA) either alone or combined for 48 h. After stimulation culture media was collected and frozen for future experiments. Adipocytes were then processed for RNA extraction or protein lysis.

Mature adipocytes used in the experiments involving Dexamethasone (Dexa) were starved over night with DMEM/Ham's F-12 (1:1, v/v) media (Thermo Fisher Scientific Inc.) supplemented with: 0.01 M HEPES pH 7.4 (Thermo Fisher Scientific Inc.), 0.5% fetal bovine serum and 100 U/ml penicillin/streptomycin (Thermo Fisher Scientific Inc.) with the addition of 500 ng/ml of Dexa (Sigma-Aldrich). The following day, adipocytes were stimulated with 100 ng/ml of IL-17A (R&D systems) for 48 h. After stimulation the adipocytes were processed for RNA extraction.

### Participant Selection

The study was approved by the ethical committee of Dubai Health Authority and Mohammed bin Rashid University of Medicine and Health Sciences Internal Review Board, Dubai, UAE. All participants provided written informed consent. Patients were recruited at Rashid Hospital, Dubai, UAE. Male and female moderate-to-severe asthmatic patients were >18 years of age, patients were diagnosed by spirometry and clinical history according to American Thoracic Society guidelines. Participants with a >20 pack-year smoking history or with a history of smoking within the last 6 months were excluded from the study.

### RNA Extraction and Quantitative Reverse Transcription Polymerase Chain Reaction (qPCR)

Extraction of total RNA from adipocytes was performed using a phenol-chloroform extraction method (RiboZol RNA extraction reagent, VWR, Leicestershire, UK), as directed in the manufacturer's instructions. Contaminating DNA was removed from 4 μg of total RNA using the AccuRT Genomic DNA Removal Kit (Applied Biological Materials, Richmond, BC, Canada), following manufacturer's protocol. Reverse transcription was performed using the 5X All-In-One Reverse Transcriptase Mastermix (ABM). The TaqMan system was used to measure gene expression for GR-α, GR-β, and GAPDH as a house keeping (Applied Biosystems, Foster City, CA, USA). Table 2 shows the list of forward and reverse primers used. The TaqMan reaction contained 2.5 μl of undiluted cDNA, 5 μl of TaqProbe 2 × qPCR Mastermix-No Dye (ABM), 0.5 μl of ready-to-use probe, and 2 μl of nuclease free H_2_O. mRNA expression of experiments using Dexamethasone was measured using a TaqMan reaction containing 1 μl of undiluted cDNA, 5 μl of TaqMan Fast Advanced Master Mix (Applied Biosystems, Foster City, CA, USA), 0.5 μl of ready-to-use probe and 2.5 μl of nuclease free H_2_O. Inflammatory marker gene targets (Table 2) were measured using EvaGreen qPCR Mastermix (ABM). The reaction was as follows: 5 μl of EvaGreen Mastermix, 2.5 μl of diluted cDNA (1/25), 0.6 μl of forward and reverse primers (10 μM) and 2.4 μl of nuclease free H_2_O. Each sample was tested in duplicates and the qPCR amplification was performed using CFX96 thermal cycler (BioRad, Hercules, CA, USA) and cycler conditions for both TaqMan and EvaGreen qPCR were preformed according to manufacturer's protocol. The ΔΔCT method was used to measure gene expression for both detection methods: amount of target = 2^−ΔΔCT^.

**Table 2:** Forward and reverse primers inflammatory markers and their oligo sequences.

**Primer name**	**Oligo sequence (5′ to 3′)**
IL-8 forward	TCTGCAGCTCTGTGTGAAGGT G
IL-8 reverse	AATTTCTGTGTTGGCGCAGTG
IL-6 forward	ACCTTCCAAAGATGGCTGAAA
IL-6 reverse	GCTCTGGCTTGTTCCCTCACTAC
IL-17A forward	GAGGACAAGAACTTCCCCCG
IL-17A reverse	CATTGCCGTGGAGATTCCAAG
TNF-α forward	CCTCTTCTCCTTCCTGATCGT
TNF-α reverse	GGTTTGCTACAACATGGGCTA
TGF-β1 forward	TACCTGAACCCGTGTTGCTCTC
TGF-β1 reverse	GTTGCTGAGGTATCGCCAGGAA
IFN-γ forward	GTTTTGGGTTCTCTTGGCTGT
IFN-γ reverse	ATGTATTGCTTTGCGTTGGAC
IL-1β forward	TACATCAGCACCTCTCAAGCA
IL-1β reverse	CCACATTCAGCACAGGACTCT
GAPDH forward	GAAGGTGAAGGTCGGAGT
GAPDH Reverse	GAAGATGGTGATGGGATTTC

### GR-α and GR-β Protein Quantification

Mature adipocytes were cultured and treated as specified above. Cell culture media was collected and placed at −80°C for future experiments. Adipocytes were washed once with 500 μl PBS, PBS was removed gently using a pipette. 1 mL of PBS was then added to each well and placed at −80°C overnight, a second freeze-thaw cycle was conducted by simply thawing the frozen cells and placing the culture plate once more at −80°C overnight. The frozen adipocyte plate is thawed the next day and the cell lysate is removed from each well and centrifuged 5 min at 5,000 × g.

From the cell lysate, the protein concentration of GR-α and GR-β were quantified using a chemiluminescence immunoassay (CLIA) and ELISA kit, for each protein, respectively. The Human GR alpha (Glucocorticoid Receptor Alpha) CLIA Kit and the Human GR beta/Glucocorticoid Receptor Beta Elisa Kit (ELISAGenie, London, UK) were used to quantify the GR proteins. Assay procedure was followed according to manufacturer's protocol, except for the following steps: standards and cell lysates were incubated in assay plate over night at 4°C, Biotin-detection antibody was incubated at room temperature for 60 min, the HRP (CLIA)/SABC (ELISA) working solution was incubated at room temperature for 30 min, the Substrate Mixture (CLIA) was incubated 5 min at room temperature and the TMB (ELISA) substrate was incubated at room temperature.

GR-α and GR-β levels in serum were measured using the following ELISA kits: Human GR alpha/Glucocorticoid Receptor Alpha Elisa Kit (ELISAGenie) and the Human GR beta/Glucocorticoid Receptor Beta Elisa Kit (ELISAGenie). Assay procedure was followed according to manufacturer's protocol.

### Cytokine Quantification

Cytokine concentrations in cell culture media secreted from treated adipocytes and serum samples was measured using a MILLIPLEX MAP Human High Sensitivity T Cell Panel—Immunology Multiplex Assay (EMDMillipore, Burlington, MA) with the following analytes: IL-4,-5,-6,-8, IFN-γ and a MILLIPLEX MAP Human TH17 Magnetic Bead Panel with the following analytes: IL2, IL-13, IL-17A, IL-17F. This assay was preformed according to manufacturer's protocol.

### Statistical Analysis

Standard statistical two-tailed *t*-tests and one-way ANOVA using Tukey's multiple comparison test were performed to test for statistical significance between data groups using GraphPad Prism 8 (GraphPad, San Diego, CA, USA). *p* < 0.05 was considered significant. Pearson correlation was used to study correlations.

## Results

### GR-α/GR-β Ratio in Lean and Obese Adipocytes

**Figure 1:**
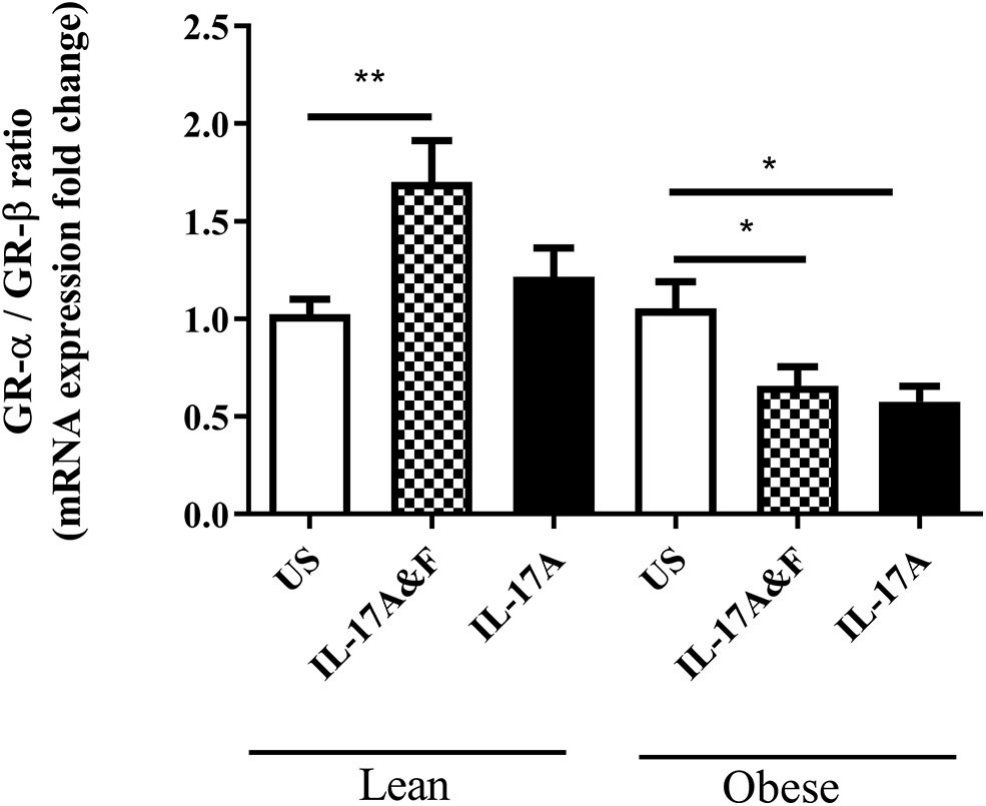
Stimulation with IL-17A & F and IL-17A alone induces changes in GR-α/GR-β mRNA ratio. Adipocytes from lean and obese subjects were stimulated with 100 ng/mL of IL-17A and IL-17F in combination or IL-17A alone for 48 h. Cells were collected and qRT-PCR analysis was performed in duplicate using TaqMan probes to assess GR-α and GR-β mRNA expression. One independent experiment performed per subject. *n* = 4 lean subjects, *n* = 3 obese subjects, One-Way ANOVA, Mean ± SE; ^*^P < 0.05, ^**^P < 0.01.

**Figure 2:**
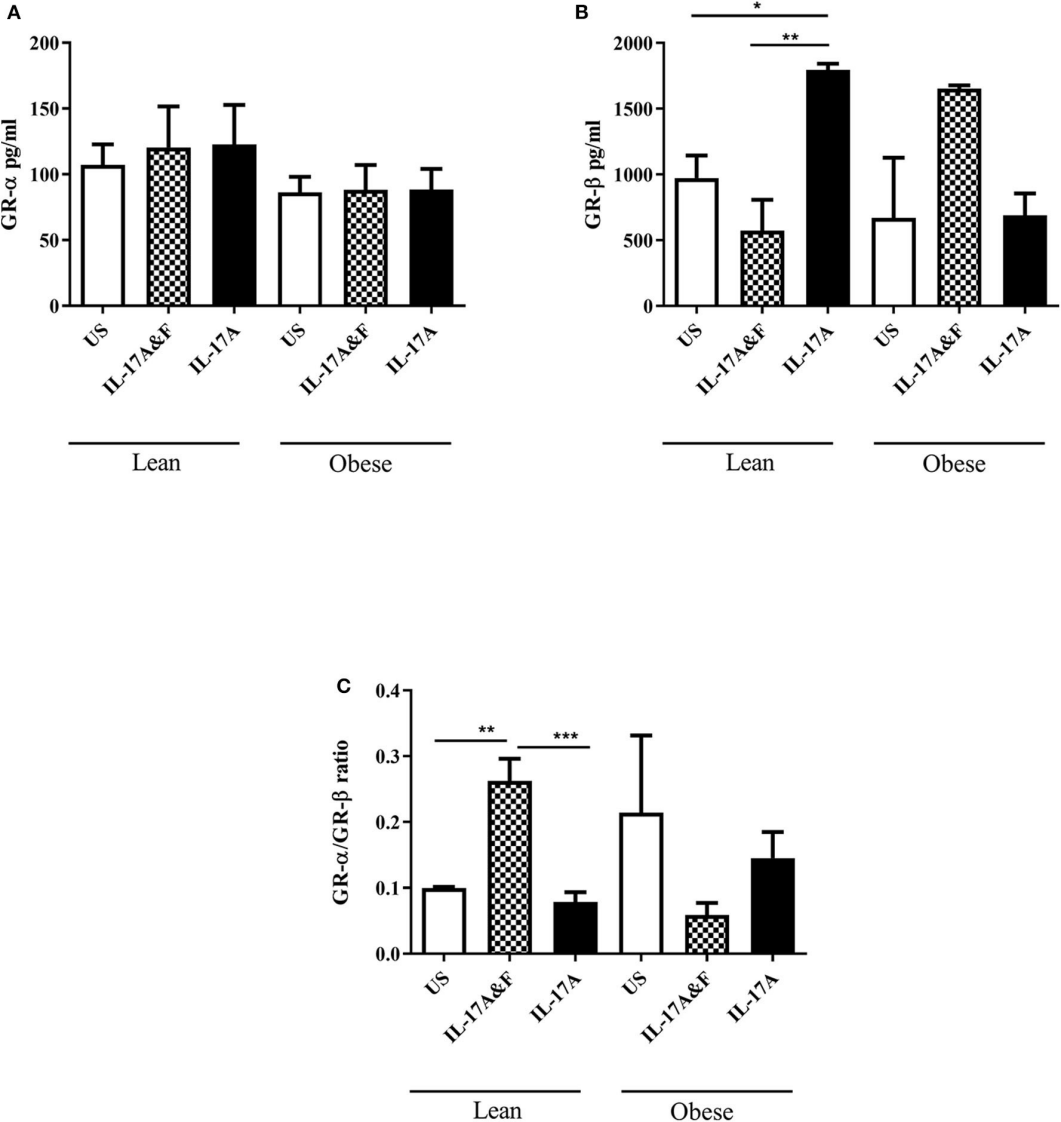
IL-17A & F and IL-17A alone induces changes in protein levels of GR-β. GR-α **(A)** and GR- β **(B)** protein expression in adipocytes from lean and obese subjects following 48 h stimulation with IL-17A&F combination or IL-17A alone **(C)** Ratio of GR-α/GR-β. *n* = 4 lean subjects, *n* = 3 obese subjects, One-way ANOVA, Mean ± SE; ^*^P < 0.05, ^**^P < 0.01, ^***^P < 0.001. Data is representative of three experiments.

Pre-adipocytes obtained from female lean (*n* = 4) and obese (*n* = 3) subjects ranging from 25 to 67 years of age (Table 1) were purchased and differentiated *in vitro*. The average BMI was 20.6 ± 2.2 kg/m^2^ and 33.4 ± 3.5 kg/m^2^ for the lean and obese adipocytes, respectively. Th17 cytokines, 100 ng/mL IL-17A and IL-7F in combination or IL-17A alone, were added for 48 h. Following stimulation, adipocytes were collected, and RNA was extracted. Adipocytes from lean subjects stimulated with IL-17A and IL-17F show a significant increase, with 2-fold change, in GR-α/GR-β ratio (*p* = 0.0057) (Figure 1). However, IL-17 stimulation of adipocytes from obese subjects shows a significant decrease in GR-α/GR-β ratio (*p* = 0.03) which has been described in asthmatic patients with steroid hyporesponsiveness (20). ELISA was used to confirm and assess protein levels of GR-α and GR-β. Cell lysates obtained from adipocytes from obese and lean subjects stimulated with IL-17A and IL-17F in combination or IL-17A alone show differential expression of GR-β (Figure 2). GR-β is highly increased (*p* = 0.03) in lean adipocytes when stimulated with IL-17A alone whereas GR-β is increased (not significant) in obese adipocytes only when stimulated with the combination of IL-17A and IL-17F compared to unstimulated cells (Figure 2B). GR-α is unchanged in response to IL-17 cytokine stimulation in both lean and obese adipocytes compared to unstimulated adipocytes (Figure 2A). Therefore, our data suggests that IL-17 cytokines may lead to an increase in GR-β mRNA and protein expression which contributes to the shift of the ratio of GR.

### Steroid Unresponsiveness in Obese Adipocytes

**Figure 3:**
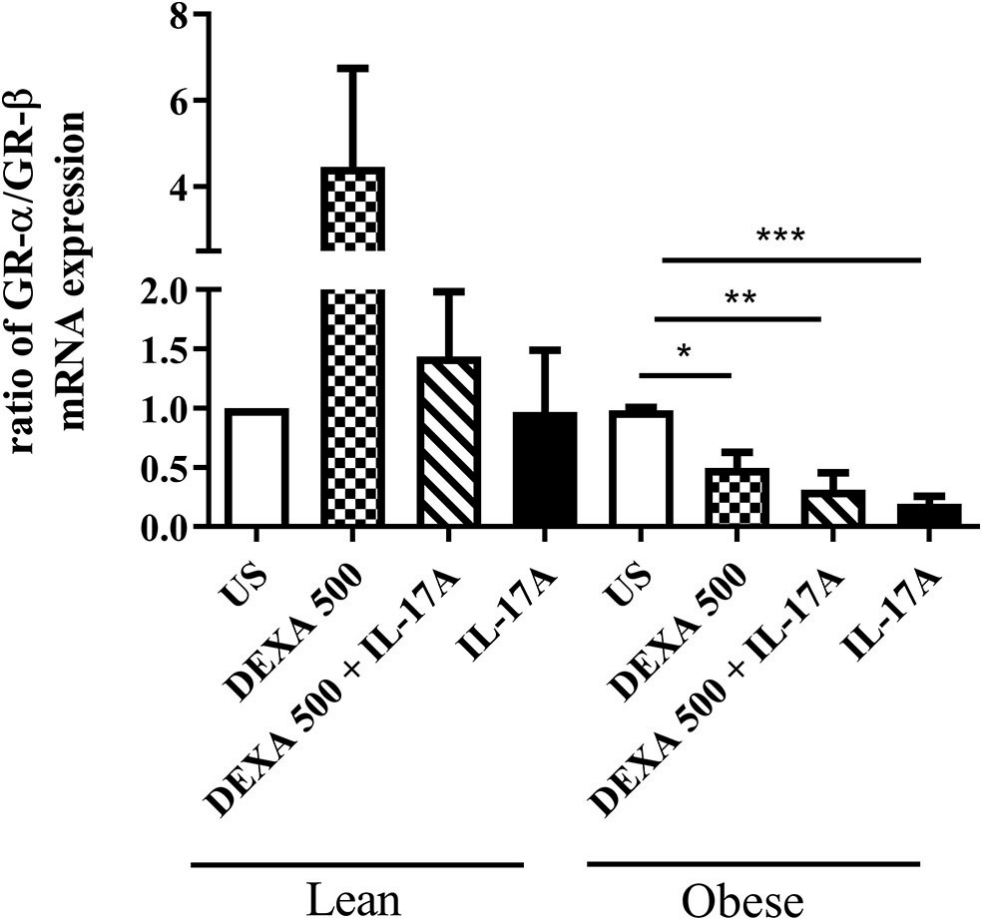
Pre-treatment with Dexamethasone followed by IL-17A stimulation induces changes in GR-α/GR-β mRNA ratio. Adipocytes from lean and obese subjects were pre-treated with 500 ng/ml dexamethasone followed by a 48 h stimulation with 100 ng/ml of IL-17A. mRNA expression of GR-α and GR-β were measured by qRT-PCR in duplicates using TaqMan probes. One independent experiment preformed per subject: *n* = 4 lean subjects, *n* = 3 obese subjects, One-Way ANOVA, Mean ± SE; ^*^P < 0.05, ^**^P < 0.01, ^***^P < 0.001.

We also sought to see the effect of IL-17 stimulation on steroid-treated adipocytes. Pre-incubation with Dexa (500 ng/mL) lead to an increase in mRNA expression of GR-α/GR-β ratio in lean adipocytes and a decreased ratio in obese adipocytes (Figure 3). This decrease in GR-α/GR-β suggests that obese adipocytes do not respond to Dexa. Interestingly, stimulation with IL-17A in pre-treated cells decreased the GR-α/GR-β ratio in both lean and obese adipocytes although not statistically significantly (Figure 3). This data suggests that IL-17 may modulate adipocyte responses to steroids and obese adipocytes are not responsive to steroid treatment.

### Changes in Cytokine Profiles in Lean and Obese Adipocytes

**Figure 4:**
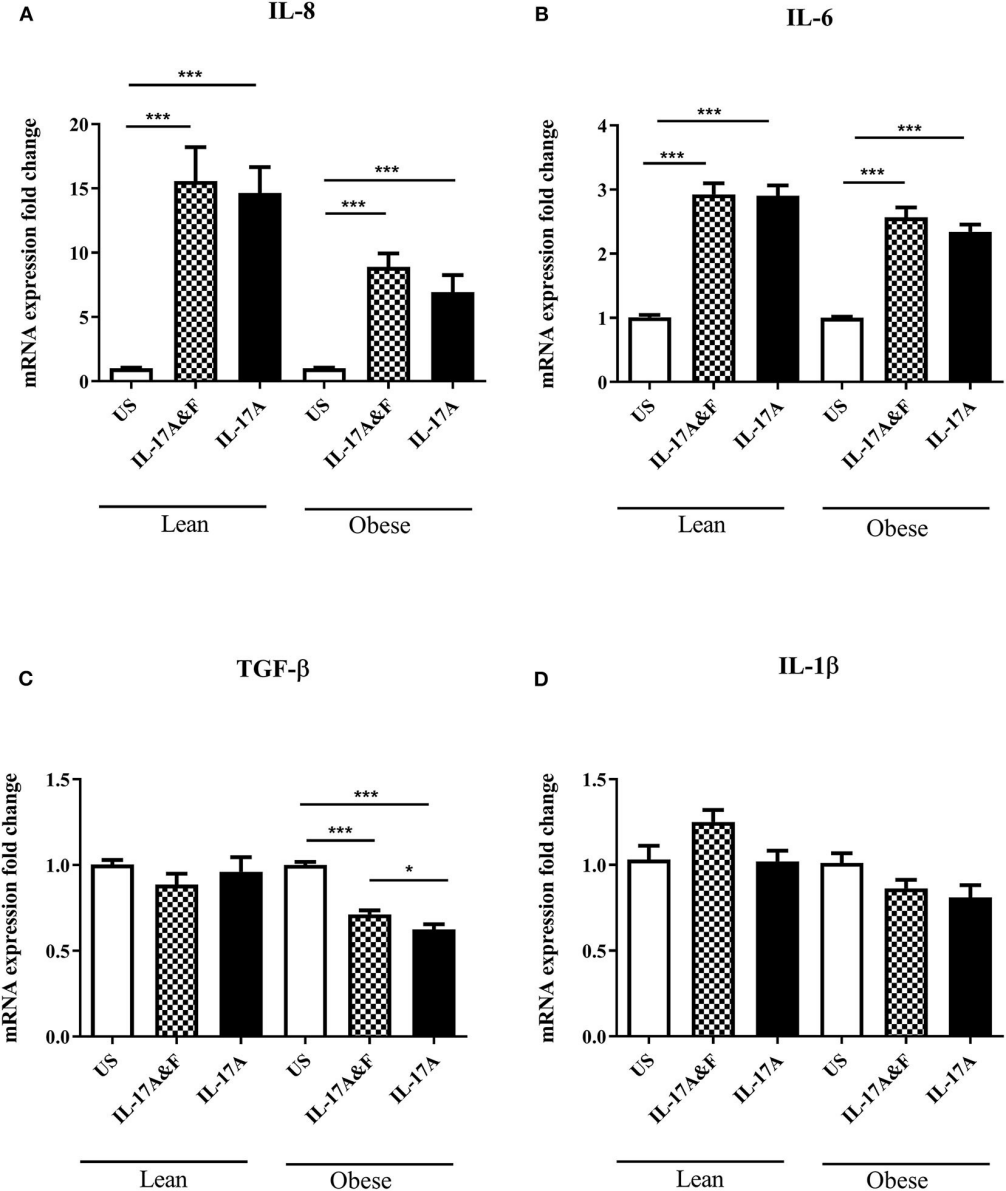
Stimulation with IL-17A&F and IL-17A alone induces changes in mRNA expression of inflammatory mediators in adipocytes from lean and obese subjects. qRT-PCR analysis of detected mRNA expression of inflammatory markers: IL-8 **(A)**, IL-6 **(B)**, TGF-β **(C)**, IL-1β **(D)** in mature adipocytes after 48 h stimulation with combination of IL-17A&F or IL-17A alone. *n* = 4 lean subjects, *n* = 3 obese subjects, One-way ANOVA, Mean ± SE; ^*^P < 0.05, ^***^P < 0.001.

**Figure 5:**
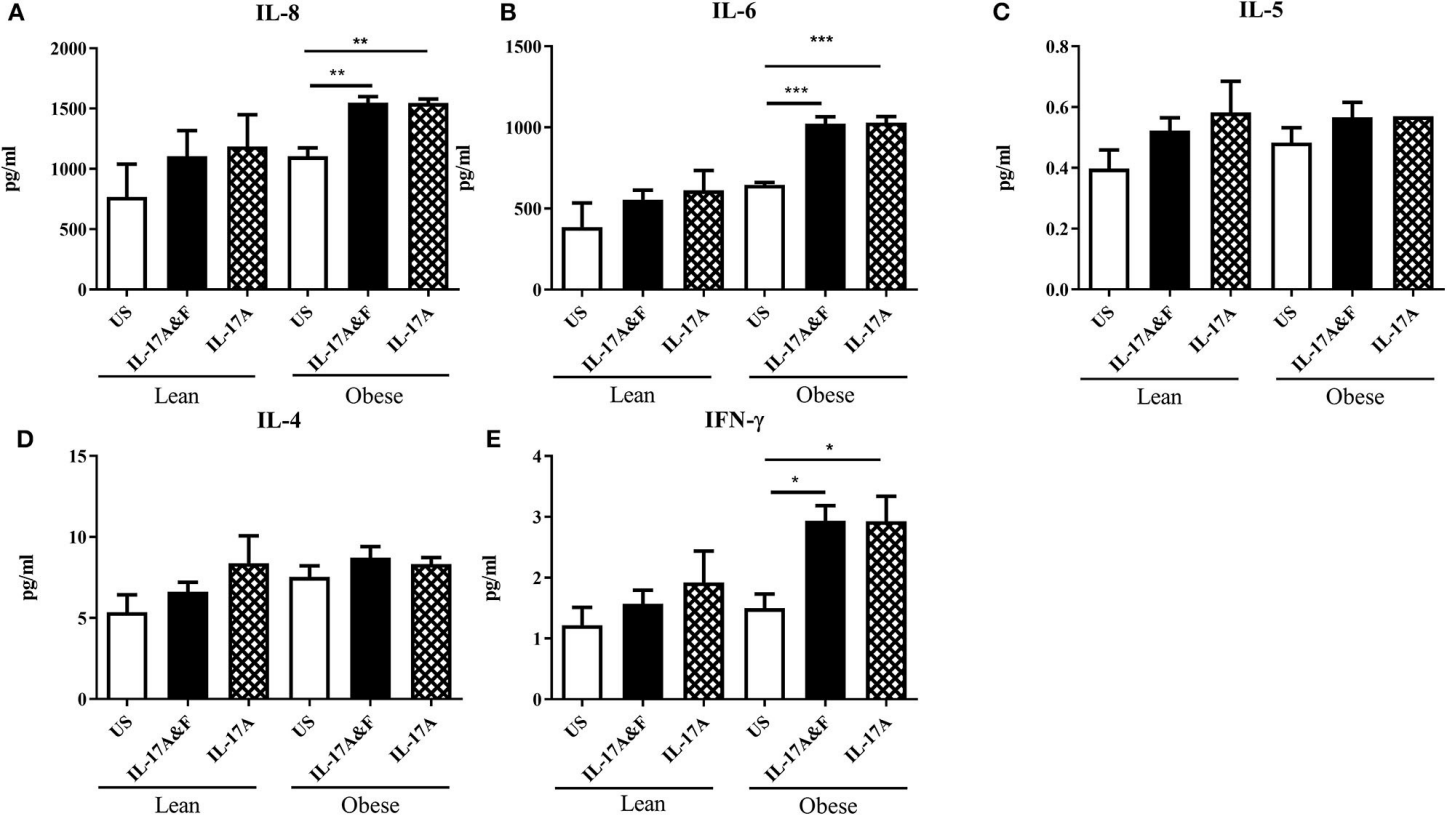
Stimulation with IL-17A&F and IL-17A alone induces changes in inflammatory cytokine profiles in adipocytes from lean and obese subjects. A multiplex assay was used to measure the levels of inflammatory cytokines: IL-8 **(A)**, IL-6 **(B)**, IL-5 **(C)**, IL-4 **(D)**, and IFN-γ **(E)** secreted by mature adipocytes after 48 h stimulation with IL-17A & F combination or IL-17A alone. One independent experiment preformed per subject. *n* = 4 lean subjects, *n* = 3 obese subjects, One-way ANOVA, Mean ± SE; ^*^P < 0.05, ^**^P < 0.01, ^***^P < 0.001.

Conditioned media was obtained from lean (*n* = 4) and obese (*n* = 3) subjects following IL-17 cytokine stimulation to assess cytokine production. mRNA expression was assessed at 48 h post-stimulation. The changes in IL-6 and IL-8 mRNA expression were observed post-stimulation in both lean and obese adipocytes (Figure 4). Interestingly, TGF-β mRNA expression was significantly decreased in obese adipocytes stimulated with IL-17 cytokines compared to unstimulated and IL-17-stimulated lean adipocytes (Figure 4C). TGF-β is an anti-inflammatory cytokine which is involved in obesity and asthma. To confirm these findings, multiplex assay was performed on conditioned media obtained 48 h post-stimulation to measure the levels of inflammatory cytokines (Figures 5A–E). At protein levels, the changes in cytokines expression were only significantly different in obese adipocytes stimulated with IL-17A and IL-17F in combination or IL-17A alone. IL-6, IL-8, and IFN-γ were significantly increased in obese adipocytes compared to unstimulated and IL-17-stimulated lean adipocytes. Our data suggests that IL-17 stimulation leads to further inflammation in adipocytes obtained from obese subjects. This is not observed in the adipocytes from lean subjects at protein level although changes were observed at mRNA level.

### GR-α/GR-β Ratio in Serum of Obese and Non-obese Asthmatics

**Figure 6:**
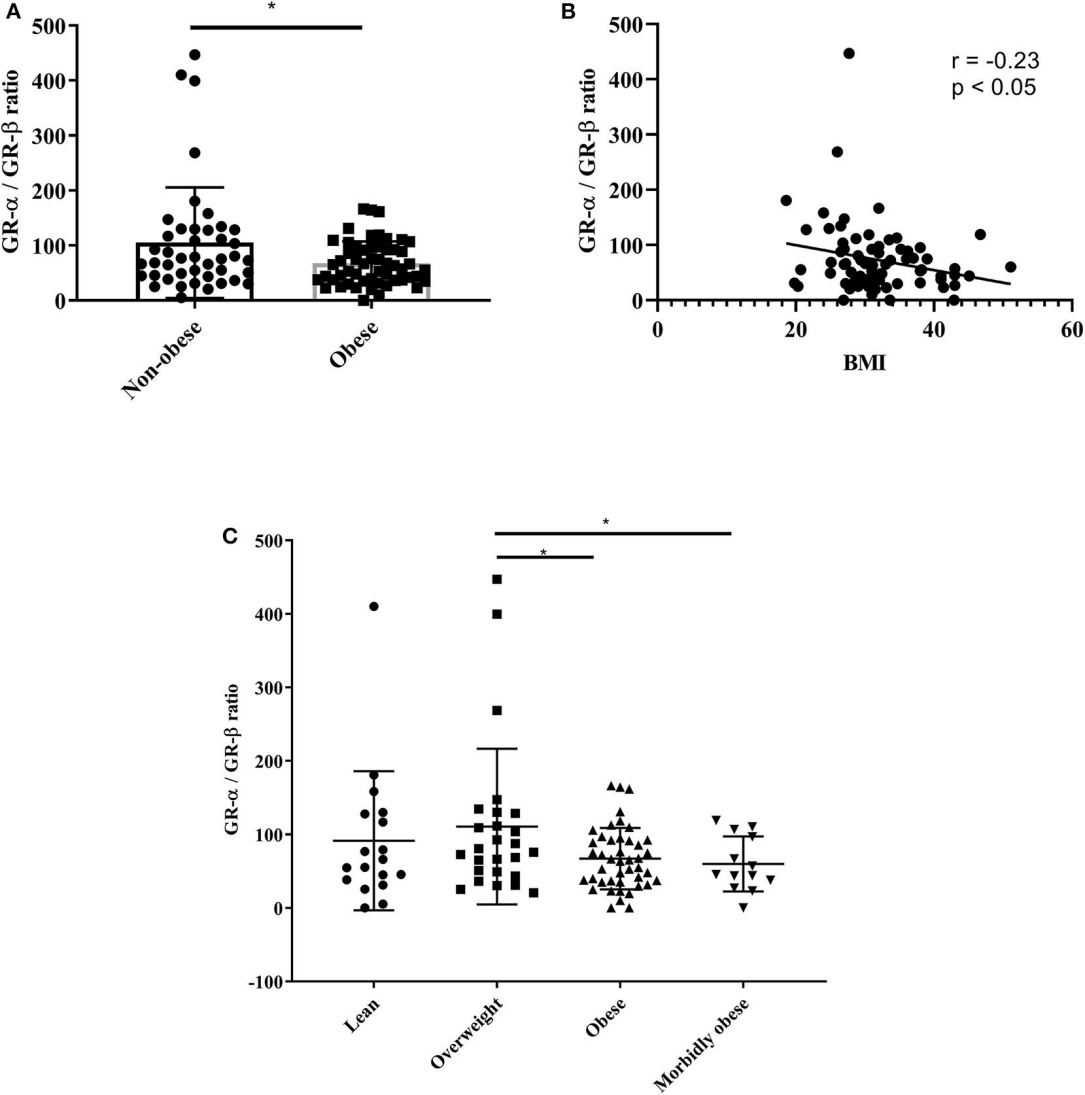
GR-α/GR-β ratio is decreased in serum of obese asthmatic subjects. Serum was obtained from lean (BMI < 25 kg/m^2^), overweight (BMI < 30 kg/m^2^), obese (BMI < 35 kg/m^2^), and morbidly obese (BMI > 35 kg/m^2^) asthmatic subjects. **(A,C)** ELISA was used to assess protein levels of GR-α and GR-β. **(B)** Pearson correlation between BMI and GR-α/GR-β ratio. *n* = 43 non-obese subjects (*n* = 18 lean, *n* = 26 overweight), *n* = 57 obese subjects (*n* = 44 obese, *n* = 13 morbidly obese), Mean ± SE; ^*^P < 0.05.

Following *in vitro* assays, we were interested to see if these findings were also observed in lean and obese asthmatics. Serum was obtained from 44 non-obese (lean and overweight) asthmatic patients and 57 obese (obese and morbidly obese) asthmatic patients. Demographic and clinical data of the patients is presented in Table 3. Table 3 shows that all lung function parameters were comparable in lean and obese moderate-to-severe asthmatics. ACT scores were 16.2 ± 0.9 and 16.4 ± 0.6 for lean and obese asthmatics, respectively. Blood eosinophils were significantly (*p* = 0.04) decreased in obese asthmatics. Previous studies have shown discrepancies in eosinophil counts in blood, sputum and biopsies. In one study in mild-to-moderate asthmatics, there was no difference in blood eosinophil's in obese and lean subjects. However, sputum eosinophils were significantly decreased in sputum and increased in bronchial submucosa (21). ELISA was performed to assess GR-α/GR-β ratio. Data revealed that obese asthmatics had a significant decrease in GR-α/GR-β ratio compared to non-obese asthmatics (Figure 6A). The non-obese asthmatics show heterogeneity in their response. Despite this, the GR-α/GR-β ratio is significantly higher than the obese asthmatics. There was a negative correlation (r = −0.23) between GR-α/GR-β ratio and BMI (*p* < 0.05) as assessed by Pearson correlation (Figure 6B) When patients were categorized further into: lean (*n* = 18), overweight (*n* = 26), obese (*n* = 44), and morbidly obese (*n* = 13) asthmatic patients, GR-α/GR-β ratio was significantly decreased in obese and morbidly obese asthmatic patients compared to overweight asthmatic patients (*p* < 0.05) (Figure 6C).

**Table 3:** Demographic and clinical data of patients.

	**Non-obese**	**Obese**
*N*	43	57
Age, year	36.3 ± 2.0	41.4 ± 2.1
BMI, kg/m^2^	25.4 ± 0.5	36.5 ± 0.8
ACT score	16.2 ± 0.9	16.4 ± 0.6
FEV_1_ (L)	2.6 ± 0.2	2.2 ± 0.1
FVC (L)	3.4 ± 0.2	2.7 ± 0.2
FEV_1_/FVC (%)	76.1 ± 1.8	79.2 ± 1.6
Differentials (%)		
• Neutrophils	55.9 ± 2.4	56.0 ± 1.6
• Lymphocytes	30.9 ± 1.9	32.6 ± 1.5
• Monocytes	7.1 ± 0.4	7.0 ± 0.4
• Eosinophils	5.8 ± 0.7	3.9 ± 0.4
• Basophils	0.6 ± 0.1	0.5 ± 0.1

*BMI, body mass index; ACT, Asthma Control Test; FEV_1_, Forced expiratory volume in 1 s; FVC: forced vital capacity. Values shown are mean ± SE*.

### IL-17F and IL-13 Protein Expression Are Increased in Obese Asthmatics

**Figure 7:**
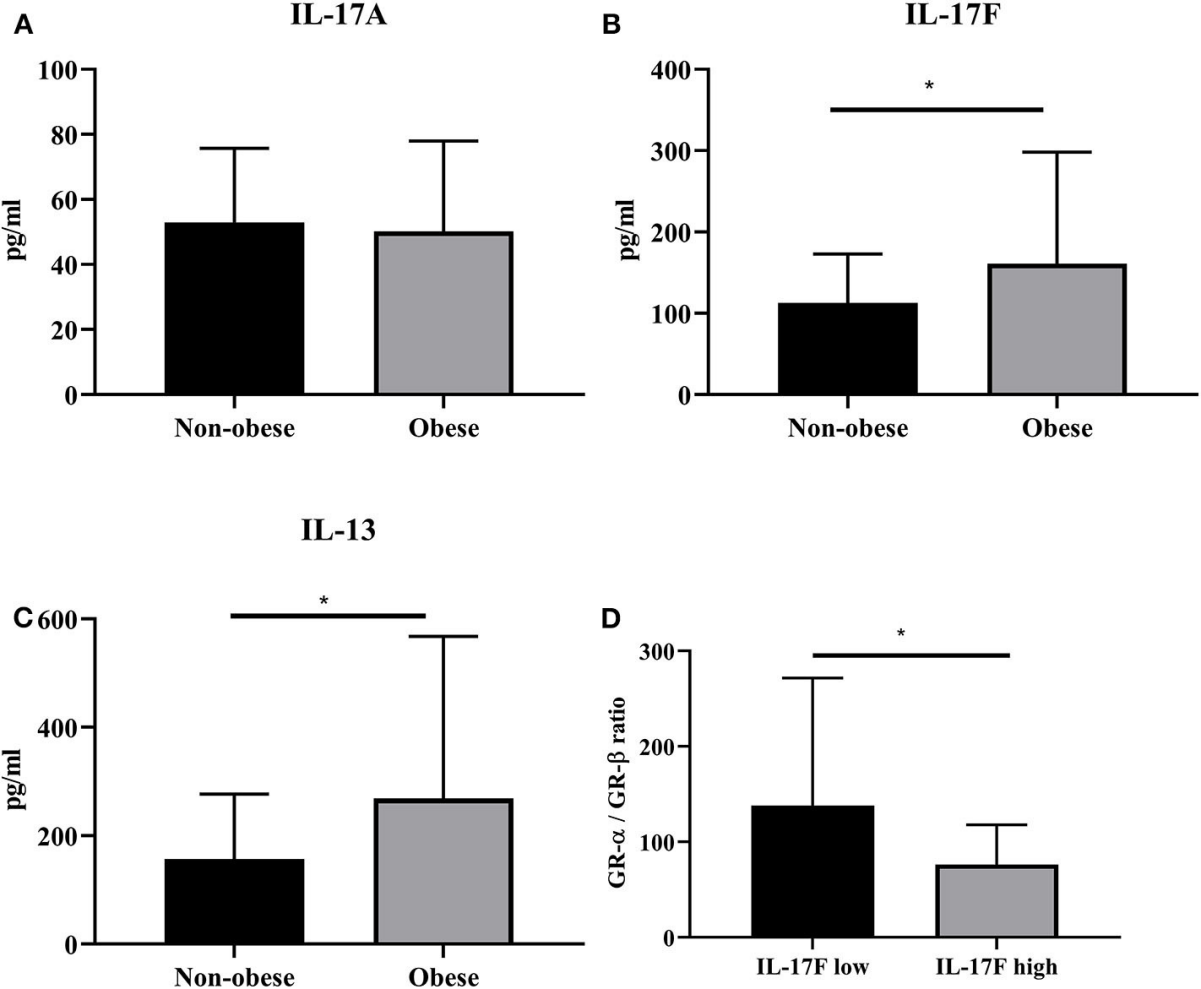
IL-17F production is increased in serum of obese asthmatics. Serum was obtained from lean (BMI < 25 kg/m^2^), overweight (BMI < 30 kg/m^2^), obese (BMI < 35 kg/m^2^), and morbidly obese (BMI > 35 kg/m^2^) asthmatic subjects. **(A–C)** ELISA was used to assess protein levels of IL-17A, IL-17F, IL-13, respectively. **(D)** GR-α/GR-β ratio in subjects with high IL-17F and low IL-17F. *n* = 43 non-obese subjects, *n* = 57 obese subjects, Mean ± SE; ^*^P < 0.05.

We analyzed the protein expression of IL-17A, IL-17F, and IL-13 in serum of obese (obese and morbidly obese) and non-obese (lean and overweight) asthmatics (Figures 7A–C). There was no difference in serum IL-17A production in non-obese compared to obese asthmatics. However, there was a statistically significant increase in IL-17F (*p* = 0.03) and IL-13 (*p* = 0.02) production in obese compared to non-obese asthmatics. We then analyzed the IL-17F levels in the lowest quartile (<90 pg/mL) and highest quartile (>180 pg/mL). Interestingly, we found a significant difference between GR-α/GR-β ratio in the lowest and highest quartile. High levels of IL-17F were associated with decreased GR-α/GR-β ratio compared to low levels of IL-17F (Figure 7D).

## Discussion

Many studies have shown positive correlation between asthma and obesity. Moreover, clinical data suggests that obese asthmatics are refractory to conventional therapy. This study demonstrates, for the first time, that IL-17 plays a role in steroid resistance through the dysregulation of GR-α and GR-β expression in adipocytes. Our data suggests that IL-17 cytokines are also involved in the upregulation of pro-inflammatory mediators in the context of obesity. These findings were further strengthened by demonstrating a negative correlation between BMI and GR-α/GR-β ratio in serum from asthmatic patients. Serum obtained from obese and morbidly obese asthmatic patients showed a significant decrease in GR-α/GR-β ratio and an increase in IL-17F and IL-13 compared to lean and overweight patients.

Although most asthmatic patients respond well to conventional therapy, 25–35% of patients show no improvement in lung function in response to inhaled corticosteroids (22). Certain subsets of asthmatic patients such as active smokers (23) and obese patients have blunted steroid responses. The dual relationship between asthma and obesity is of interest as studies indicate that obesity does not necessarily cause asthma but may be a risk factor for the development and the severity of asthma. In both adults and children, the obese asthma phenotype tends to lead to more severe symptoms akin to the severe asthma phenotype. Of interest, 60% of severe asthmatic patients are obese (24). A body of literature has shown that one of the major players in steroid resistance in severe asthma is the defect in GR-α and thus dysregulation of the GR-α/GR-β ratio. Since this mechanism has been described in severe asthma, we were interested to see if this was also the case in obese asthmatics. We found that the ratio was decreased in obese asthmatics compared to non-obese asthmatics. This was statistically significant in obese and morbidly obese compared to overweight asthmatics. Interestingly, a correlation analysis revealed a statistically significant negative correlation between BMI and GR-α/GR-β ratio. This is of clinical relevance as BMI may predict the steroid responsiveness of asthmatic patients.

Having established that the ratio of GR-α/GR-β is altered in obese asthmatics, we were interested to see what mediators could be involved in this dysregulation. Obesity is associated with increased markers of inflammation in serum and adipose tissue in obese people with asthma. In the obese state, the adipose tissue is infiltrated with proinflammatory cytokines and adipokines. This led to the hypothesis that proinflammatory responses in the adipose tissue may lead to asthma. One of the major proinflammatory cytokines involved in obesity as well as asthma is IL-17A.The role of Th17 cells in obesity is relatively unexplored but evidence of accumulation of Th17 cells in a mouse model of diet-induced obesity has been described (25). Studies have suggested that obesity predisposes to the expansion of Th17 cells via IL-6 which may in turn exacerbate inflammatory conditions such as multiple sclerosis (26). However, the role of Th17 cells and its cytokines in obesity and, in particular, in obese asthma remains largely unknown. Therefore, we sought to study the role of IL-17A and IL-17F in steroid hyporesponsiveness. Adipocytes from lean and obese subjects were cultured in the presence of IL-17A and IL-17F in combination and IL-17A alone. IL-17A alone was used due to the overwhelming amount of literature that suggests a role for this proinflammatory cytokine in obesity. Adipocytes obtained from lean subjects stimulated in the presence of 100 ng/mL of IL-17A and IL-17F showed a large increase in GR-α/GR-β at mRNA level. This finding is in line with the potential dual role of IL-17 cytokines, where IL-17 may play a role in tissue homeostasis. However, adipocytes obtained from obese subjects which were stimulated with IL-17A and IL-17F showed a decrease in GR-α/GR-β ratio at mRNA level. Results obtained from protein differed in responses. Lean and obese adipocytes stimulated with IL-17 cytokines showed an increase in the negative regulator, GR-β. Although most studies on steroid resistance report a dysregulation with GR-α, our results showed no change in GR-α protein. Nonetheless, the overall effect of IL-17 cytokines is a decrease in the GR-α/GR-β ratio in both lean and obese adipocytes. Moreover, it would seem that obese adipocytes respond more to IL-17F. Very little amount of literature is available on the role of IL-17F in obesity as it is simply described as a closely related cytokine of IL-17A. Interestingly, in serum obtained from asthmatics patients, IL-17F protein expression is increased in obese and morbidly obese patients compared to lean and overweight patients whereas IL-17A is unchanged. The serum levels of Th17 cytokines are consistent with a previous study which showed that a healthier diet led to decreases in IL-17F but not IL-17A (27). Moreover, the levels of IL-17 were much higher than IL-17A. We also found an increase in IL-13 protein expression in serum of obese asthmatics compared to lean asthmatics. IL-13 is a pro-inflammatory cytokine involved in allergic asthma. IL-17A has been shown to enhance IL-13 activity (28). In mice, IL-13 treatment induced airway hyperresponsiveness and led to increased numbers of IL-17-producing CD4+ T cells (29). Increase in IL-13 has been reported in general obesity (30). Serum levels of IL-13 positively correlate with BMI (31). However, its role in the obese asthma phenotype is unknown and further investigations should be done to determine its exact function. In an animal model of obesity, it has been reported that BAL and serum IL-17A levels were not affected by the type of diet. However, pulmonary IL-17 mRNA levels were increased in high-fat diet animals compared to chow fed animals. Moreover, flow cytometry revealed an increase in IL-17A producing cells in the lungs (32). These results indicate that the changes in Th17 cytokines are observed locally within the lungs but obesity does not lead to increased systemic inflammation in asthma models. This warrants further investigations of IL-17-producing cells in the lung or adipose tissue of obese asthmatics compared to lean asthmatics. Demographic and clinical data for the lean and obese moderate-to-severe asthmatics revealed that lung function was similar in both populations whereas blood eosinophil's were decreased in obese asthmatics, This is in line with previous literature which has shown a negative correlation between BMI and blood eosinophil's in a population with high eosinophil's (33). The obese asthma phenotype is a complex phenotype where not all obese asthmatics share similar clinical features. However, studies have shown that clinical features that are common in patients with high BMI are late onset asthma, less airway eosinophil's and reduced atopy (34).

Adipose tissue, which is mainly composed of adipocytes, is a major source of proinflammatory cytokines such as IL-6, IL-8, IL-10, TNF-α, and IL-18 (35) thus linking obesity and inflammation. Due to their proinflammatory properties, IL-17 cytokines may be involved in the association between obesity and inflammation. Therefore, we were interested in examining the role of IL-17 in the production of inflammatory mediators by adipocytes obtained from obese and lean subjects. Adipocytes were stimulated with IL-17A and IL-17F for 48 h. Stimulation with IL-17 led to an increase in IL-6 and IL-8 mRNA. At protein level, this change was only observed in adipocytes from obese subjects. This is of interest as small adipocytes in lean individuals have been shown to promote homeostasis whereas large adipocytes from obese individuals promote inflammation and are involved in the recruitment of macrophages (36). This highlights a differential function for adipocytes in relation to body weight. In this study we found that obese adipocytes respond to IL-17 through the release of pro-inflammatory cytokines, which will lead to exaggerated inflammatory responses. Interestingly, steroid-treated obese adipocytes showed a decrease in GR-α/GR-β ratio which was further decreased in the presence of IL-17A. IL-17A was able to decrease the GR-α/GR-β ratio in steroid-treated lean adipocytes which did respond to Dexa. This finding suggests that IL-17 is capable of altering responses to steroid. In a recent study on neutrophilic inflammation in asthma, it was shown that Dexa and IL-17A in combination synergistically induced the expression of the neutrophil promoting cytokine CSF3 and Dexa alone failed to alleviate neutrophilic inflammation (37).

In conclusion, our data suggest that IL-17 cytokines are involved in the inflammatory response seen in obese subjects. Moreover, IL-17 is involved in the dysregulation of glucocorticoid receptors which may explain steroid hyporesponsiveness commonly described in obese asthmatics. BMI can be used a predictor for steroid responsiveness. IL-17F and IL-13, which is increased in obese asthmatics, may be involved in the dysregulation of GR-α/GR-β ratio.

## Data Availability Statement

All datasets generated for this study are included in the article/supplementary material.

## Ethics Statement

The studies involving human participants were reviewed and approved by Dubai Health Authority Mohammed bin Rashid University of Medicine and Health Sciences Internal Review Board. The patients/participants provided their written informed consent to participate in this study.

## Author Contributions

SA designed experiments, analyzed the samples, and contributed to the manuscript preparation. MG performed experiments, analyzed the samples, and contributed to the manuscript preparation. RR contributed to the manuscript preparation. AM performed ELISA experiments. LS collected samples from the patients. BM collected samples from the patients. QH contributed to the design of the experiments and manuscript preparation. All authors read and approved final version of the manuscript.

## Conflict of Interest

The authors declare that the research was conducted in the absence of any commercial or financial relationships that could be construed as a potential conflict of interest.

## References

[1] Kelly T Yang W Chen CS Reynolds K He J. Global burden of obesity in 2005 and projections to 2030. Int J Obes. (2008) 32:1431–7. 10.1038/ijo.2008.102 18607383

[2] Khaodhiar L McCowen KC Blackburn GL. Obesity and its comorbid conditions. Clin Cornerstone. (1999) 2:17–31. 10.1016/S1098-3597(99)90002-9 10696282

[3] Mohanan S Tapp H McWilliams A Dulin M. Obesity and asthma: pathophysiology and implications for diagnosis and management in primary care. Exp Biol Med (Maywood). (2014) 239:1531–40. 10.1177/1535370214525302 24719380 PMC4230977

[4] Peters MC McGrath KW Hawkins GA Hastie AT Levy BD Israel E. Plasma interleukin-6 concentrations, metabolic dysfunction, and asthma severity: a cross-sectional analysis of two cohorts. Lancet Respir Med. (2016) 4:574–84. 10.1016/S2213-2600(16)30048-0 27283230 PMC5007068

[5] Straczkowski M Kowalska I Nikolajuk A Dzienis-Straczkowska S Szelachowska M Kinalska I. Plasma interleukin 8 concentrations in obese subjects with impaired glucose tolerance. Cardiovasc Diabetol. (2003) 2:5. 10.1186/1475-2840-2-5 12793907 PMC162167

[6] Hotamisligil GS Spiegelman BM. Tumor necrosis factor alpha: a key component of the obesity-diabetes link. Diabetes. (1994) 43:1271–8. 10.2337/diabetes.43.11.1271 7926300

[7] Nishimura S Manabe I Nagai R. Adipose tissue inflammation in obesity and metabolic syndrome. Discov Med. (2009) 8:55–60. 19788868

[8] Kern PA Ranganathan S Li C Wood L Ranganathan G. Adipose tissue tumor necrosis factor and interleukin-6 expression in human obesity and insulin resistance. Am J Physiol Endocrinol Metab. (2001) 280:E745–51. 10.1152/ajpendo.2001.280.5.E745 11287357

[9] McArdle MA Finucane OM Connaughton RM McMorrow AM Roche HM. Mechanisms of obesity-induced inflammation and insulin resistance: insights into the emerging role of nutritional strategies. Front Endocrinol. (2013) 4:52. 10.3389/fendo.2013.00052 23675368 PMC3650620

[10] Ruddy MJ Wong GC Liu XK Yamamoto H Kasayama S Kirkwood KL. Functional cooperation between interleukin-17 and tumor necrosis factor-alpha is mediated by CCAAT/enhancer-binding protein family members. J Biol Chem. (2004) 279:2559–67. 10.1074/jbc.M308809200 14600152

[11] Cosmi L Liotta F Maggi E Romagnani S Annunziato F. Th17 cells: new players in asthma pathogenesis. Allergy. (2011) 66:989–98. 10.1111/j.1398-9995.2011.02576.x 21375540

[12] Sumarac-Dumanovic M Stevanovic D Ljubic A Jorga J Simic M Stamenkovic-Pejkovic D. Increased activity of interleukin-23/interleukin-17 proinflammatory axis in obese women. Int J Obes. (2009) 33:151–6. 10.1038/ijo.2008.216 18982006

[13] Ramakrishnan RK Al Heialy S Hamid Q. Role of IL-17 in asthma pathogenesis and its implications for the clinic. Expert Rev Respir Med. (2019) 13:1057–68. 10.1080/17476348.2019.1666002 31498708

[14] Al-Ramli W Prefontaine D Chouiali F Martin JG Olivenstein R Lemiere C. T(H)17-associated cytokines (IL-17A and IL-17F) in severe asthma. J Allergy Clin Immunol. (2009) 123:1185–7. 10.1016/j.jaci.2009.02.024 19361847

[15] Tashiro H Shore SA. Obesity and severe asthma. Allergol Int. (2019) 68:135–42. 10.1016/j.alit.2018.10.004 30509734 PMC6540088

[16] Mosen DM Schatz M Magid DJ Camargo CA Jr. The relationship between obesity and asthma severity and control in adults. J Allergy Clin Immunol. (2008) 122:507–11.e6. 10.1016/j.jaci.2008.06.024 18774387

[17] Forno E Lescher R Strunk R Weiss S Fuhlbrigge A Celedon JC. Decreased response to inhaled steroids in overweight and obese asthmatic children. J Allergy Clin Immunol. (2011) 127:741–9. 10.1016/j.jaci.2010.12.010 21377042 PMC3056233

[18] Goleva E Covar R Martin RJ Leung DYM. Corticosteroid pharmacokinetic abnormalities in overweight and obese corticosteroid resistant asthmatics. J Allergy Clin Immunol Pract. (2016) 4:357–60.e2. 10.1016/j.jaip.2015.11.013 26795244 PMC4789098

[19] Sobande PO Kercsmar CM. Inhaled corticosteroids in asthma management. Respir Care. (2008) 53:625–33. discussion 33–4. 18426616

[20] Vazquez-Tello A Halwani R Hamid Q Al-Muhsen S. Glucocorticoid receptor-beta up-regulation and steroid resistance induction by IL-17 and IL-23 cytokine stimulation in peripheral mononuclear cells. J Clin Immunol. (2013) 33:466–78. 10.1007/s10875-012-9828-3 23160983

[21] van der Wiel E Ten Hacken NH van den Berge M Timens W Reddel HK Postma DS. Eosinophilic inflammation in subjects with mild-to-moderate asthma with and without obesity: disparity between sputum and biopsies. Am J Respir Crit Care Med. (2014) 189:1281–4. 10.1164/rccm.201310-1841LE 24832748

[22] Martin RJ Szefler SJ King TS Kraft M Boushey HA Chinchilli VM. The predicting response to inhaled corticosteroid efficacy (PRICE) trial. J Allergy Clin Immunol. (2007) 119:73–80. 10.1016/j.jaci.2006.10.035 17208587 PMC2872157

[23] Tomlinson JE McMahon AD Chaudhuri R Thompson JM Wood SF Thomson NC. Efficacy of low and high dose inhaled corticosteroid in smokers versus non-smokers with mild asthma. Thorax. (2005) 60:282–7. 10.1136/thx.2004.033688 15790982 PMC1747368

[24] Schatz M Hsu JW Zeiger RS Chen W Dorenbaum A Chipps BE Phenotypes determined by cluster analysis in severe or difficult-to-treat asthma. J Allergy Clin Immunol. (2014) 133:1549–56. 10.1016/j.jaci.2013.10.006 24315502

[25] Winer S Paltser G Chan Y Tsui H Engleman E Winer D. Obesity predisposes to Th17 bias. Eur J Immunol. (2009) 39:2629–35. 10.1002/eji.200838893 19662632

[26] Ji Z Wu S Xu Y Qi J Su X Shen L. Obesity promotes EAE through IL-6 and CCL-2-mediated T cells infiltration. Front Immunol. (2019) 10:1881. 10.3389/fimmu.2019.01881 31507583 PMC6718738

[27] Han YY Forno E Brehm JM Acosta-Pérez E Alvarez M Colón-Semidey A. Diet, interleukin-17, and childhood asthma in Puerto Ricans. Ann Allergy Asthma Immunol. (2015) 115:288–93.e1. 10.1016/j.anai.2015.07.020 26319606 PMC4721241

[28] Hall SL Baker T Lajoie S Richgels PK Yang Y McAlees JW. IL-17A enhances IL-13 activity by enhancing IL-13-induced signal transducer and activator of transcription 6 activation. J Allergy Clin Immunol. (2017) 139:462–71.e14. 10.1016/j.jaci.2016.04.037 27417023 PMC5149451

[29] Kinyanjui MW Shan J Nakada EM Qureshi ST Fixman ED. Dose-dependent effects of IL-17 on IL-13-induced airway inflammatory responses and airway hyperresponsiveness. J Immunol. (2013) 190:3859–68. 10.4049/jimmunol.1200506 23509346

[30] Schmidt FM Weschenfelder J Sander C Minkwitz J Thormann J Chittka T. Inflammatory cytokines in general and central obesity and modulating effects of physical activity. PLoS ONE. (2015) 10:e0121971. 10.1371/journal.pone.0121971 25781614 PMC4363366

[31] Martínez-Reyes CP Gómez-Arauz AY Torres-Castro I Manjarrez-Reyna AN Palomera LF Olivos-García A. Serum levels of interleukin-13 increase in subjects with insulin resistance but do not correlate with markers of low-grade systemic inflammation. J Diabetes Res. (2018) 2018:7209872. 10.1155/2018/7209872 29675435 PMC5841096

[32] Mathews JA Wurmbrand AP Ribeiro L Neto FL Shore SA. Induction of IL-17A precedes development of airway hyperresponsiveness during diet-induced obesity and correlates with complement factor D. Front Immunol. (2014) 5:440. 10.3389/fimmu.2014.00440 25309539 PMC4164008

[33] Sunadome H Matsumoto H Izuhara Y Nagasaki T Kanemitsu Y Ishiyama Y. Correlation between eosinophil count, its genetic background and body mass index: the nagahama study. Allergol Int. (2020) 69:46–52. 10.1016/j.alit.2019.05.012 31272903

[34] Baffi CW Winnica DE Holguin F. Asthma and obesity: mechanisms and clinical implications. Asthma Res Pract. (2015) 1:1. 10.1186/s40733-015-0001-7 27965756 PMC4970376

[35] Fantuzzi G. Adipose tissue, adipokines, and inflammation. J Allergy Clin immunol. (2005) 115:911–9; quiz 20. 10.1016/j.jaci.2005.02.023 15867843

[36] Thomas D Apovian C. Macrophage functions in lean and obese adipose tissue. Metabolism. (2017) 72:120–43. 10.1016/j.metabol.2017.04.005 28641779 PMC5516622

[37] Ouyang S Liu C Xiao J Chen X Lui AC Li X. Targeting IL-17A/glucocorticoid synergy to CSF3 expression in neutrophilic airway diseases. JCI insight. (2020) 5:e132836. 10.1172/jci.insight.132836 32051346 PMC7098787

